# Mitochondrial genome of *Salix cardiophylla* and its implications for infrageneric division of the genus of *Salix*

**DOI:** 10.1080/23802359.2020.1827065

**Published:** 2020-10-05

**Authors:** Xiong Chen, Li Zhang, Yuan Huang, Fuwei Zhao

**Affiliations:** a School of Life Sciences, Yunnan Normal University, Kunming, Yunnan, P. R. China; b Ministry of Ecology and Environment, Nanjing Institute of Environmental Sciences, Nanjing, Jiangsu, P. R. China

**Keywords:** Mitochondrial genome, *Salix cardiophylla*, phylogenomic analysis

## Abstract

*Salix cardiophylla* was a member of the genus of *Salix* in family Salicaceae with unique morphological traits, and once recognized as a separate genus, *Toisusu* Kimura. Here, we sequenced and assembled the complete mitochondrial genome of *S. cardiophylla*, which was 735,173 bp in length, including 56 genes, 28 protein-coding genes, 3 rRNA genes, 25 tRNA genes, and one large inverted repeat regions with length of 13,603 bp. Phylogenetic analysis based on 26 mitochondrial CDS confirmed that *S. cardiophylla* is a member of *Salix*, and support its merge into *Salix* in aspect of our new insights on mitogenome phylogenomics.

*Salix cardiophylla* Trautv. & C.A.Mey. is deciduous tree up to 20 m high found in north to central Honshu and southern Kurils of Japan, growing along the gravel river. *Salix cardiophylla* belongs to the genus *Salix* (family Salicaceae), because of its unique reproductive organs, such as the pendulous female catkins and the deciduous styles, it was once treated as a separate genus, i.e., *Toisusu* Kimura (Barkalov and Kozyrenko 2014). The genus *Toisusu* was then merged into *Salix* afterwards based on plastid and nuclear sequence evidence (Azuma et al. 2000; Ohashi 2001; Chen et al. 2010). However, merge of *Toisusu* into *Salix* based on phylogentic inference of mitochondrial sequence is lack.

Here, we used a modified CTAB method (Sahu et al. 2012), the complete mitochondrial genomic DNA was extracted from young leaves of *S. cardiophylla* which grown in Honshu, Japan (36°N, 138°E). Voucher specimens were stored in the Herbarium of Kunming Institute of Botany (Accession no: T. Tanaka s.n., cult.). The genomic library for Illumina paired-end (PE) sequencing was constructed using the Illumina Hiseq X Ten sequencer. A total of ca. 31.2 million generated and this sequence has been deposited in the NCBI Sequence Read Archive (SRA) with accession number SRR12534676. MiSeq raw reads were assembled via NOVOPlasty v2.7.2 with a *k*-mer of 39 (Dierckxsens et al. 2016). Taking the *S. paraflabellaris* mitogenome as the reference sequence (Chen et al. 2019; Huang et al. 2019), the annotation of genome assembled was carried out with Geneious v2020.1.1 software (Kearse et al. 2012).

The high-quality annotated mitogenome sequences of *S. cardiophylla* were deposited in the GenBank database under the accession No. MT806745. The mitochondrial genome of *S. cardiophylla* was assembled as a closed-circular molecule of 7,35,173 bp in length. It contains 28 protein-coding genes, including ones for NADH dehydrogenase (nad3, 4, 4 L, 6, 7, 9), succinate dehydrogenase (sdh4), apocytochrome b (cob), cytochrome c oxidase (cox1, 2, 3), ATP synthase (atp1, 4, 6, 8, 9), cytochrome c biogenesis (ccmB, C, Fn, Fc), ribosomal proteins (rpl2, 16 and rps1, 3, 4, 7, 12), maturase and membrane transporter (matR). Meantime, the genome contains 25 tRNA genes, and 3 rRNA genes are annotated. The overall base composition is 27.5% A, 22.5% C, 22.3% G, and 27.7% T, and the AT content is higher than GC content. Furthermore, we used the Repeat Finder implemented in Geneious and identified two large repeats with length of 13,603 bp. Similar to the mitogenome of *S. paraflabellaris*, no genes or protein-coding sequences harbored in the two repeats.

To explore the controversial systematic position of *S. cardiophylla*, four *Salix* species and two outgroup *Populus* species with mitogenome sequence were downloaded from GenBank (Kersten et al. 2016; Wei et al. 2016; Ye et al. 2017; Choi et al. 2017; Chen et al. 2019; Huang et al. 2019). Ultimately, 26 mitochondrial CDS shared by all these seven species were aligned by software MAFFT v7.47 (Katoh and Standley 2013). After manual adjustment, the maximum likelihood (ML) tree was constructed using the software IQ_TREE 1.6.2 (Nguyen et al. 2015) based on an array with the aligned seven sequences, branch support was estimated by bootstrap value and SH-like approximate likelihood ratio (SHAlrt) (Guindon et al. 2010) with 10,000 replicates under HKY + F model according to Bayesian information criterion by the software Model Finder (Kalyaanamoorthy et al. 2017). Our ML revealed that five *Salix* species formed a robust monophyletic clade, confirmed that *S. cardiophylla* is a member of *Salix* and support the treatment of merging it back into *Salix* based on our new insights from mitogenome phylogenomic inference (Figure 1). This work provides a valuable source of data for the study of the controversial systematic position of *S. cardiophylla*.

**Figure 1.**
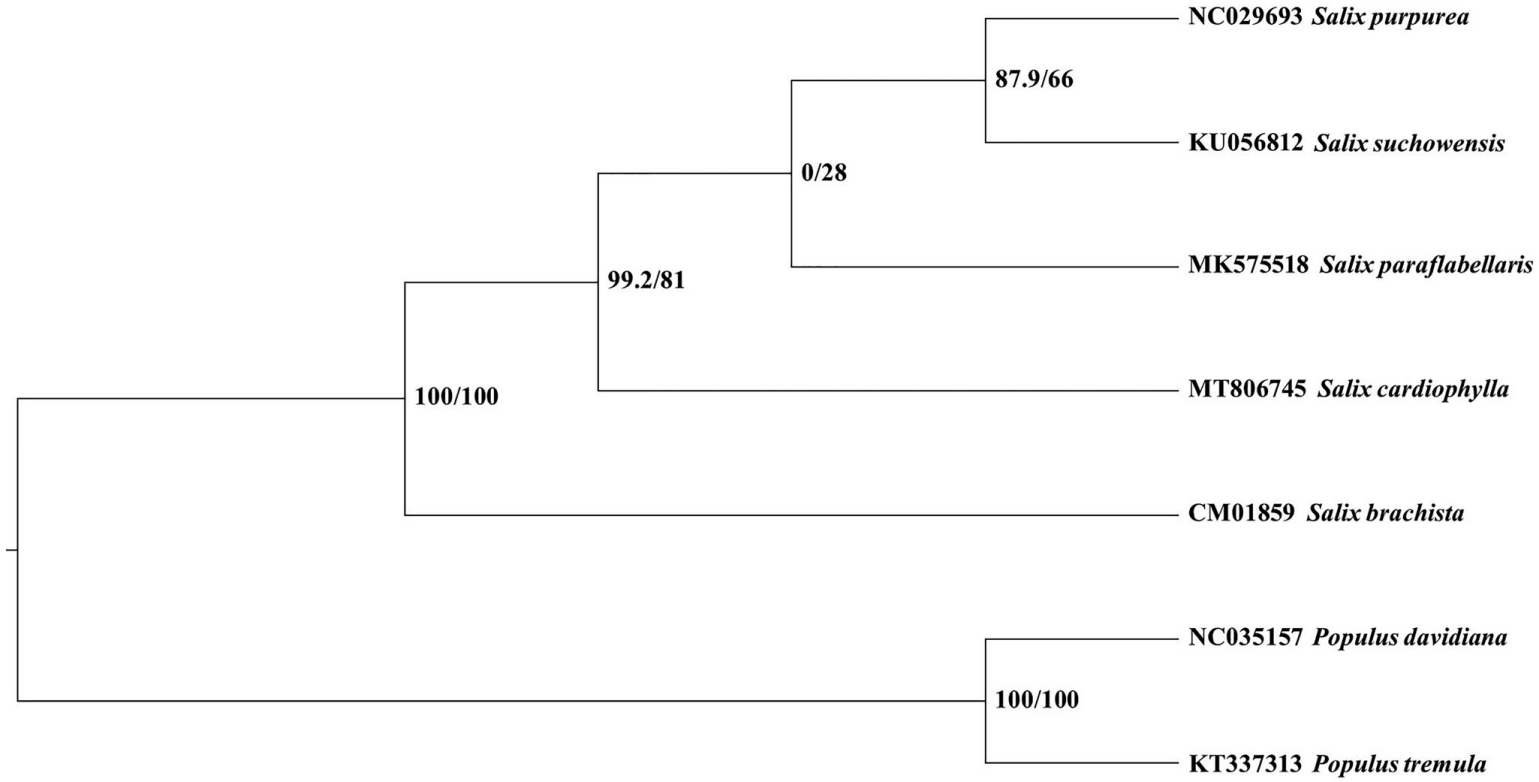
ML phylogenetic tree of *S. cardiophylla* and six Salicaceae species based on CDS shared by all these seven mitochondrial complete genome, branch supports values were reported as SH-aLRT/UFBoot.

## Data Availability

The data that support the findings of this study are openly available in NCBI at https://www.ncbi.nlm.nih.gov, reference number MT806745, SRR12534676.
